# An Aminosteroid Derivative Shows Higher In Vitro and In Vivo Potencies than Gold Standard Drugs in Androgen-Dependent Prostate Cancer Models

**DOI:** 10.3390/cancers15113033

**Published:** 2023-06-02

**Authors:** Donald Poirier, Jenny Roy, René Maltais, Cindy Weidmann, Étienne Audet-Walsh

**Affiliations:** 1 Endocrinology and Nephrology Unit, CHU de Québec Research Center-Université Laval, Pavillon CHUL, Québec, QC G1V 4G2, Canada; jenny.roy@crchudequebec.ulaval.ca (J.R.); rene.maltais@crchudequebec.ulaval.ca (R.M.); cindy.weidmann@crchudequebec.ulaval.ca (C.W.); etienne.audet-walsh@crchudequebec.ulaval.ca (É.A.-W.); 2 Department of Molecular Medicine, Faculty of Medicine, Université Laval, Québec, QC G1V 0A6, Canada

**Keywords:** prostate cancer, LAPC-4 xenograft, aminosteroid, endoplasmic reticulum stress, cholesterol, fatty acid

## Abstract

**Simple Summary:**

Prostate cancer (PC) remains fourth in terms of new cases of cancer in the world, and most deaths are caused by metastatic PC, for which limited therapeutic options are available. Faced with a lack of effective treatment, it is, therefore, urgent to dedicate more effort to the development of new innovative drugs to reverse this trend. Having developed an original molecule with promising antiproliferative activity on various cancer cell types, namely the aminosteroid derivative RM-581, its evaluation was extended to other PC models, the androgen-dependent LAPC-4 cells, and tumors. The most important result was its ability to completely block tumor growth in mice when given orally at a dose as low as 3 mg/kg. Another important result was its non-toxicity in mice during a 28-day experiment and a 7-week dose-escalation study, whose last RM-581 dose was 720 mg/kg given orally, suggesting a favorable safety window for this new promising drug.

**Abstract:**

The aminosteroid derivative RM-581 blocks with high potency the growth of androgen-dependent (AR^+^) prostate cancer VCaP, 22Rv1, and LAPC-4 cells. Notably, RM-581 demonstrated superior antiproliferative activity in LAPC-4 cells compared to enzalutamide and abiraterone, two drugs that exhibited a synergistic effect in combination with RM-581. These findings suggest that RM-581 may have an action that is not directly associated with the hormonal pathway of androgens. Furthermore, RM-581 completely blocks tumor growth in LAPC-4 xenografts when given orally at 3, 10, and 30 mg/kg in non-castrated (intact) nude mice. During this study, an accumulation of RM-581 was observed in tumors compared to plasma (3.3–10 folds). Additionally, the level of fatty acids (FA) increased in the tumors and livers of mice treated with RM-581 but not in plasma. The increase was greater in unsaturated FA (21–28%) than in saturated FA (7–11%). The most affected FA were saturated palmitic acid (+16%), monounsaturated oleic acid (+34%), and di-unsaturated linoleic acid (+56%), i.e., the 3 most abundant FA, with a total of 55% of the 56 FA measured. For cholesterol levels, there was no significant difference in the tumor, liver, or plasma of mice treated or not with RM-581. Another important result was the innocuity of RM-581 in mice during a 28-day xenograft experiment and a 7-week dose-escalation study, suggesting a favorable safety window for this new promising drug candidate when given orally.

## 1. Introduction

Important therapeutic innovations have emerged in the last decade, significantly improving the global survival rate of certain cancers [1,2]. Prostate cancer (PC) is among these diseases. However, despite this progress, it continues to rank fourth in terms of new cancer cases worldwide, with approximately 1.41 million reported cases. Additionally, it remains the fifth cause of death in men due to cancer, resulting in over 375,000 deaths in 2020 [3,4,5,6]. Most of those deaths are caused by metastatic PC, for which limited therapeutic options are available [7,8]. Given the lack of effective treatment options, there is an urgent need to increase investment in the development of new promising molecules to reverse this trend. The arrival of innovative drugs to treat resistant and advanced PC is obviously highly desirable.

**Figure 1:**
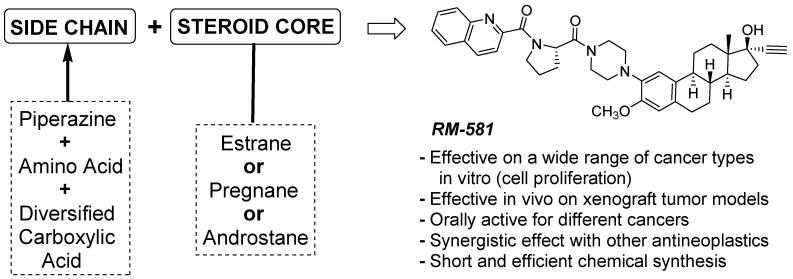
RM-581 is representative of a new generation of steroidal anticancer agents that induce endoplasmic reticulum stress and apoptosis.

Over the last years, our research group has designed, synthesized, pharmacokinetically characterized, and evaluated, in vitro and in vivo, a new family of aminosteroids as anticancer agents [9,10,11,12,13,14,15,16,17,18,19,20,21,22]. These anticancer molecules, developed through a phenotypic approach, were optimized by solid-phase and classic chemical synthesis. This process led to the identification of crucial key structure-activity relationship elements, particularly in relation to the lateral side chain moiety at C2 and the steroidal scaffold [9,10,11,12,15,16,19,20]. These findings highlight the importance of finely modulating the biological activity of these molecules. The broad and selective action of aminosteroids on multiple cancer cell lines (pancreatic, breast, prostate, leukemia, and ovarian) highlights their large anticancer therapeutic potential [13,19,20]. Although their molecular target(s) remain to be clearly identified, these steroids were found to induce a pro-apoptotic action through the aggravation of endoplasmic reticulum stress with an associated disturbance of lipid homeostasis in different cell lines [14,17,21,22]. Built initially around a 17α-ethynyl-androsterone (androstane) backbone [9,10,11], the last generation of this molecular platform retains the key side chain but includes a mestranol (estrane) core [18,19,20]. RM-581 (Figure 1) represents the lead candidate of this new family of anticancer agents.

After developing an original lead candidate molecule with promising antiproliferative activity, namely the aminosteroid derivative RM-581, it is necessary to extend its evaluation to other cancer models. To this end, and in addition to previous in vitro and in vivo evaluations of RM-581 on the androgen-independent PC represented by the PC-3 cell line [22], the androgen-dependent VCaP, 22Rv1, and LAPC-4 cell lines were used for a screening study. Among them, the LAPC-4 cell line was selected to perform a series of in vitro assays (cell proliferation and drug combination) followed by in vivo assays (xenograft and dose-limit experiments). The effects of RM-581 treatment on lipids (cholesterol and fatty acids) have also been investigated and are presented herein.

## 2. Materials and Methods

### 2.1. Cell Lines

Human prostate cancer VCaP and 22Rv1 cell lines were obtained from ATTC, while LAPC-4 cells were kindly provided by Robert E. Reiter from the University of California (Los Angeles, CA, USA).

### 2.2. PCa Cell Viability Proliferation (Screening Assays with VCaP, 22Rv1, and LAPC-4)

VCaP (4 × 10^4^ cells/well) were seeded in 96-well plates in DMEM media supplemented with 10% fetal bovine serum (FBS), penicillin, streptomycin, and sodium pyruvate. 22Rv1 cells (2 × 10^3^ cells/well) and LAPC-4 cells (6 × 10^3^ cells/well) were seeded in 96-well plates in RPMI media supplemented with 10% FBS, penicillin, streptomycin, and sodium pyruvate. The cells were incubated at 37 °C in a 5% CO_2_ humidified atmosphere for 24 h before treatment with multiple concentrations of RM-581 dissolved in dimethyl sulfoxide (DMSO). The concentrations used were between 0.01 μM and 50 μM. Treatment lasted for 3 days for all the cell lines except VCaP cells, which were treated for 7 days due to slower proliferation rates. At the end of the assay, 20 μL of MTS (Cell Titer 96 Aqueous, Promega, Nepean, ON, Canada) were added to each well, and the mixture was incubated for 4 h at 37 °C. The plates were subsequently analyzed at 490 nm using a Tecan M-200 microplate reader (Männedorf, Switzerland). The concentration required to inhibit 50% of proliferation (IC_50_) was calculated using the QuestGraph IC50 calculator (https://www.aatbio.com/tools/ic50-calculator (accessed on 28 May 2023)).

### 2.3. LAPC-4 Cell Viability Proliferation and Combination Index Assays

LAPC-4 cells (1 × 10^4^ cells/well) were seeded in triplicate in 96-well plates in IMDM (Iscove’s modified Dulbecco’s medium) (90 μL) supplemented (*v*/*v*) with 5% fetal FBS, 1% L-glutamine, 1% insulin, and 1% penicillin/streptomycin. The cells were incubated at 37 °C in a 5% CO_2_ humidified atmosphere for 24 h. RM-581 or reference drugs were dissolved in DMSO, and the stock solutions (10 mM) were diluted with culture media to obtain the desired final concentration when using 10 μL in each well. After 6 days of incubation, the cell proliferation was determined by adding 10 µL of MTS followed by incubation at 37 °C for 4 h. Plates were then analyzed at 490 nm using a Tecan M-200 microplate reader (Männedorf, Switzerland). Using GraphPad Prism 7 software, the IC_50_ values were calculated. The values represent the average of two independent experiments performed in triplicate. According to the Chou–Talalay method [23] and using the following formula, the CI (combination) was calculated.

$$CI=\frac{{\left(D\right)}_{1}}{{{(D}_{X})}_{1}}+\frac{{\left(D\right)}_{2}}{{{(D}_{X})}_{2}}$$

Denominators (D_X_)_l_ and (D_X_)_2_ are the doses of individual drugs required to achieve a given effect level, and numerators (D)_1_ and (D)_2_ are the concentrations of each drug present in combination to trigger the same effect level. When the drugs interact additively, CI = 1, but a CI < 1 indicates a synergistic interaction, and a CI > 1 indicates an antagonistic effect.

### 2.4. Apoptosis Characterization by Flow Cytometry

This experiment was performed with LAPC-4 cells (3 × 10^5^) following the protocol previously reported for PC-3 cells [22]. After the detection of annexin-V and propidium iodide fluorescence, dual parametric dot plots allowed us to calculate the percentage of each cell type: (1) non-apoptotic live cells (annexin-V-negative/PI-negative) in the lower left quadrant; (2) early apoptotic cells (annexin-V-positive/PI-negative) in the lower right quadrant; (3) late apoptotic cells (annexin-V-positive/PI-positive) in the upper right quadrant; and (4) necrotic cells (annexin-V-negative/PI-positive) in the upper left quadrant.

### 2.5. Gene Expression Study by qPCR Analysis

LAPC-4 cells (3 × 10^5^) were treated with RM-581 (1, 2, and 5 µM) at different times (6, 18, 24, and 36 h) and the expression of various genes was determined as previously reported [22,24]. Primer sequences used for the analysis of each gene are reported in Table S3 (Supplementary Materials).

### 2.6. Effect of RM-581 in LAPC-4 Xenograft Experiment

The xenograft experiment was approved by the Université Laval Animal Care Committee (Comité de Protection des Animaux de l’Université Laval, Québec, QC, Canada) and carried out in an animal facility approved by the Canadian Council on Animal Care (CCAC). Homozygous male Balb/c athymic nude mice (~42 days old, weighing approximately 28 g) were obtained from Charles River Inc. (Saint-Constant, QC, Canada) and housed (4 to 5) in vinyl micro-isolated ventilated cages equipped with air lids. Cages were kept in laminar airflow hoods and maintained in pathogen-limiting conditions. The animals were housed under a controlled environment at 22 ± 3 °C with 50 ± 20% relative humidity and light set at 12 h/day (light on at 07:15) during the acclimatization and study periods. Sterile water and rodent food (Rodent Diet #T.2018.15, Harlan Teklad, Madison, WI, USA) were provided ad libitum.

After an acclimatization period of 5 days, the mice were inoculated into both flanks by subcutaneous (SC) injection of 4 × 10^6^ LAPC-4 cells (Passage 14) in 0.1 mL of growth medium containing 50% Matrigel (BD Biosciences, Mississauga, ON, Canada) via a 2.5-cm long 22-gauge needle. Two weeks later, mice that developed tumors were randomized according to tumor volume, divided into 7 groups (Gr-1 to Gr-8), and treated over a period of 28 days. A bilateral orchiectomy was performed under isoflurane-induced anesthesia for Gr-1 but not for Gr-2 to Gr-8. Both Gr-1 (CTX control (CTL), 9 mice, 16 tumors) and Gr-2 (intact CTL, 9 mice, 18 tumors) were treated with 0.1 mL of the vehicle alone (8% DMSO/92% propylene glycol (PG) administered by gavage (PO). Gr-3 (30 mg/kg/PO, 10 mice, 19 tumors) received 0.843 mg of RM-581. Gr-4 (10 mg/kg/PO, 10 mice, 20 tumors) received 0.281 mg of RM-581. Gr-5 (3 mg/kg/PO, 10 mice, 18 tumors) received 0.084 mg of RM-581. Gr-6 (60 mg/kg/SC, 9 mice, 18 tumors) received 1.686 mg of abiraterone. Gr-7 (60 mg/kg/PO, 9 mice, 17 tumors) received 1.686 mg of abiraterone acetate. Gr-8 (4 mg/kg/IP, 9 mice, 17 tumors) received 0.112 mg of docetaxel.

RM-581 and abiraterone acetate were administered six days a week PO; abiraterone was administered six days a week SC; and docetaxel was administered twice a week by intraperitoneal (IP) injection. RM-581 was first dissolved in DMSO, and thereafter we added PG to obtain the appropriate concentration in the vehicle fluid (8% DMSO/92% PG). Abiraterone acetate and abiraterone were dissolved in 0.3% hydroxypropyl cellulose in saline (0.9% NaCl), while docetaxel was first dissolved in DMSO, and thereafter we added phosphate-buffered saline (PBS) to obtain the proper concentration in the carrier fluid (0.5% DMSO/99.5% PBS). All solutions were prepared one day prior to the initiation of treatment and stored at 4 °C under constant agitation.

The mice were weighed at the start and end of the protocol. Tumor area (mm^2^) was calculated twice a week using two perpendicular diameters (L and W), which were recorded using a caliper, and the formula L/2 × W/2 × π. The area measured on the first day of treatment was fixed at 100%. At the end of the studies and 3 h after the last treatment, the mice were terminally anesthetized, and their final body weights were measured. Tumors, liver, kidney, and total prostate were collected from mice during necropsy, while blood was collected by cardiac puncture. Tissues and plasma were immediately frozen at −80 °C until analysis.

### 2.7. Dosage of RM-581 in Tumors and Plasma

A procedure developed at the Bioanalytical Service of the CHU de Québec-Research Center-Université Laval (Québec, QC, Canada) and previously published [21,22,25], was used to measure by liquid chromatography–mass spectrometry/mass spectrometry (LC-MS/MS) analysis the concentration of RM-581 tumors and treated mice.

### 2.8. Tolerated Dose of RM-581 in Mice

The maximum dose of RM-581 that does not cause toxic effects in animals (weight loss below 20%, inability to eat or drink, abnormal posture, movement, or vocalization) was determined in mice. It is important to note that animal death is not an accepted endpoint for CCAC. RM-581 was first dissolved in DMSO, and PG was added to obtain a final 8% concentration of co-solvent DMSO. The injected volume was 0.1 mL, but the number of injections ranged from 1, 2, or 3 to obtain the expected dose. Each week for 6 consecutive weeks, an increased dose (60, 120, 240, 360, 480, 600, or 720 mg/kg) of RM-581 was administered by gavage (PO), and the mice were weighed and observed for 7 days to notice any signs of toxicity. The protocol was applied to four groups (Gr) divided as follows: Gr-1: 5 female mice received the vehicle only; Gr-2: 5 male mice received the vehicle only; Gr-3: 5 female mice treated with RM-581; and Gr-4: 5 male mice treated with RM-581.

### 2.9. Fatty Acid Determination in LAPC-4 Tumors

A solution of chloroform and methanol (2:1, *v*/*v*) was used to extract lipids from 80 mg of tissue from LAPC-4 tumors, as previously described [26]. The fatty acid profile was next determined by gas chromatography (GC), coupled with a flame ionization detector (FID), using phosphatidylcholine C21:0 (Avanti Polar Lipids) as an internal standard. The GC model 5890 was from Agilent Technologies (Mississauga, ON, Canada). The capillary column used was a HP-88 (100 m × 0.25 mm, 0.2 mm, Agilent Technologies), and the carrier gas was helium at 1.5 mL/min. A total of 56 fatty acids were identified according to their retention time, using fatty acid mixtures as standards [27].

### 2.10. Cholesterol Determination in LAPC-4 Cells, Tumors, Liver, and Plasma

A cholesterol quantification kit provided by Sigma-Aldrich (Catalog Number MAK043) [28] was used following the manufacturer’s instructions to determine the concentration of total cholesterol, free cholesterol, and cholesteryl ester in samples. The cholesterol contents were measured in samples from two different protocols. Firstly, in LAPC-4 cells treated (18, 36, and 72 h) or not with RM-581 (2 µM) or atorvastatin (10 µM). Secondly, in tumor, liver, and plasma obtained at the end of the LAPC-4 xenograft experiment (control group and the group treated with RM-581 at 10 mg/kg/PO).

### 2.11. Cholesterol Determination in Plasma from LAPC-4 Xenograft Experiments

Total cholesterol, triglycerides, cholesterol-HDL (high-density lipoproteins), cholesterol-LDL (low-density lipoproteins), cholesterol non-HDL, and the ratio of total cholesterol/cholesterol-HDL were determined in the plasma of mice treated PO with RM-581 (3, 10, and 30 mg/kg) or abiraterone acetate (60 mg/kg). Analyses were performed at the CHU de Québec/L’Enfant-Jésus (Medical Hospital Laboratory; Project N°: 9014625056) using a Cobas Lipid Panel from Roche.

### 2.12. Histopathology of Mouse Prostate, Kidneys, and Liver from LAPC-4 Xenograft Experiments

Tissue samples from xenograft experiments were fixed in neutral buffered 10% formalin and processed for routine paraffin embedding. Tissue sections (3 µm) were stained with hematoxylin and eosin (HE) and histologically observed by an independent pathologist. Digital images at 20× magnification of each slide were obtained using the slide scanner NanoZoomer 2.0-HT (Hamamatsu, Bridgewater, NJ, USA). Whole-section images were visualized using the software NDP View (Hamamatsu, Bridgewater, NJ, USA).

### 2.13. Statistical Analysis

Statistical significance was determined according to the Duncan–Kramer multiple-range test [29]. Other differences were evaluated using a *t*-test. *p* values of less than 0.05 were considered statistically significant.

## 3. Results

### 3.1. Antiproliferative Activity of RM-581 on Prostate Cancer Cell Lines

The aminosteroid derivative RM-581 has already demonstrated its antiproliferative activity on three cancer cell lines widely used in PC research [30]: DU-145, PC-3, and LNCaP. The effective concentrations that inhibit 50% of cell proliferation (IC_50_) for these cell lines are as follows: 4.4 µM for DU-145, 1.2 µM for PC-3, and 1.2 µM for LNCaP [22]. To extend the evaluation to androgen-dependent cell lines whose androgen receptor (AR) was present or not mutated, similar to in the LNCaP cells, RM-581 was first tested on VCaP, 22Rv1, and LAPC-4 cell lines, providing IC_50_ values of 5.78, 1.38, and 0.56 μM, respectively (Table 1). In this cell viability screening assay, the LAPC-4 cell line provided the best antiproliferative activity and was selected for additional studies. These PC-cells express a wild-type AR, are tumorigenic, and were initially obtained from lymph node metastasis in a patient with stage D disease [31].

**Figure 2:**
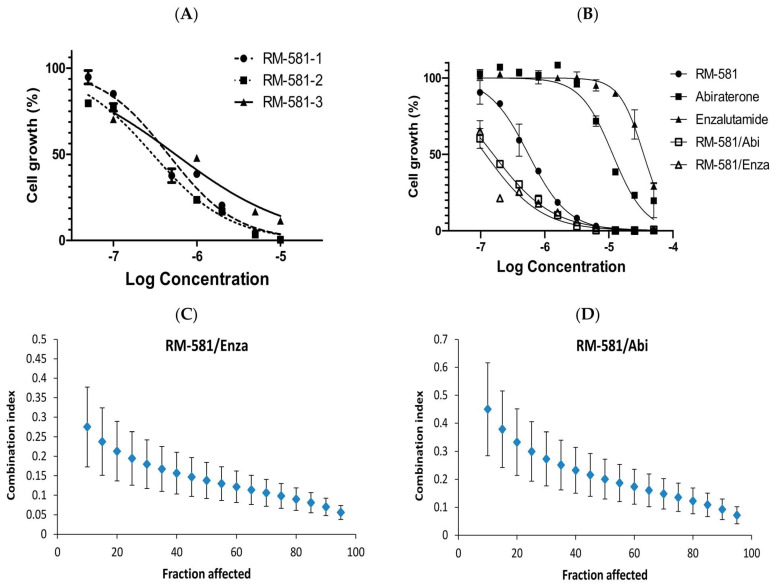
Efficacy of RM-581 in reducing LAPC-4 cell proliferation. (**A**) Concentrations of RM-581 that reduced cell growth by 50% (IC_50_ values) after six days of treatment. IC_50_ = 0.45, 0.30, and 0.55 µM. The mean of three experiments in triplicate (IC_50_ = 0.43 ± 0.13 µM). (**B**) IC_50_ values of RM-581 (0.56 µM), enzalutamide (34.8 µM), abiraterone (11.5 µM), RM-581/enzalutamide in proportions 1:1 (0.12 µM), and RM-581/abiraterone in proportions 1:1 (0.16 µM). (**C**,**D**) The combination of RM-581 with enzalutamide or abiraterone produces a synergic effect in LAPC-4 cells. The Chou–Talalay method [23] was used to calculate the combination index (CI) values. Drug synergy, addition, and antagonism are defined by combination index (CI) values less than 1.0, equal to 1.0, or greater than 1.0, respectively.

After the selection of the LAPC-4 cell line, the antiproliferative activity of RM-581 and known cytotoxic drugs was assessed. In this cell viability assay using MTS, the IC_50_ values ranged from 0.30 to 0.55 µM (Figure 2A). In comparison, two drugs used in PC treatment, the antiandrogen enzalutamide [32,33,34] and androgen biosynthesis inhibitor abiraterone [35,36,37] weakly inhibited cell proliferation with IC_50_ values of 34.8 and 11.5 μM, respectively (Figure 2B). However, when a 50:50 combination of RM-581 with each of these two drugs was tested, IC_50_ values of 0.12 and 0.16 μM were obtained for enzalutamide and abiraterone, respectively. In comparison, the IC_50_ of RM-581 was 0.56 μM. Moreover, using the Chou–Talalay methodology [23], combination indices (CI) of 0.11 and 0.15 (Figure 2C,D) were obtained. This finding indicated a synergistic effect of RM-581 with both anticancer drugs. This result also suggests that RM-581 has a mechanism of action that is different from those of enzalutamide and abiraterone.

**Figure 3:**
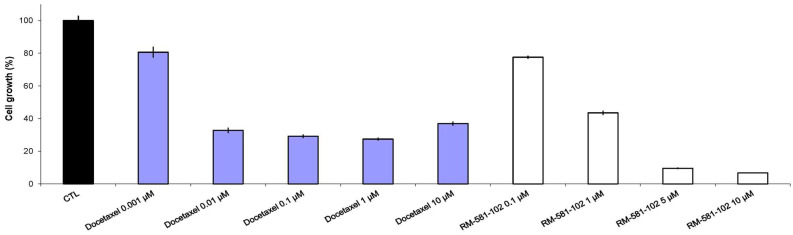
Efficacy of docetaxel and RM-581 in reducing LAPC-4 cell proliferation after six days of treatment. This is the result of four experiments in triplicate providing the same result but performed under four slightly different conditions (Figure S2 in Supplementary Materials for representations of each curve): (1) Standard protocol with the change of medium; (2) with an initial washing of the cells; (3) with an initial washing of the cells and without change of medium; and (4) without an initial washing of the cells and with the change of the medium. For docetaxel, the IC_50_ value was not calculated because of the plateau. For RM-581, IC_50_ = 0.51, 0.47, and 0.73 µM. Mean IC_50_ = 0.57 ± 0.14 µM.

The antiproliferative activity of RM-581 was also compared to that of docetaxel, a cytotoxic agent established in the last decades as a gold standard for the treatment of metastatic castration-resistant PC [38]. This antineoplastic works by disrupting the normal function of microtubules and thereby stopping cell division [39]. As observed in PC-3 cells [22], the cell growth decreased between 0.001 and 0.01 µM of docetaxel to quickly reach a plateau whose values peak at around 25% between 0.01 and 10 µM (Figure 3). The same result was also observed when slight modifications were made to the cell culture conditions, including changes in the medium as well as variations with or without an initial washing of the cells (Figure S2, Supplementary Materials). Unlike docetaxel, RM-581 produced dose-dependent inhibition at the tested concentrations with a mean value of IC_50_ = 0.57 ± 0.14 µM (Figure 3), similar to those already obtained.

**Figure 4:**
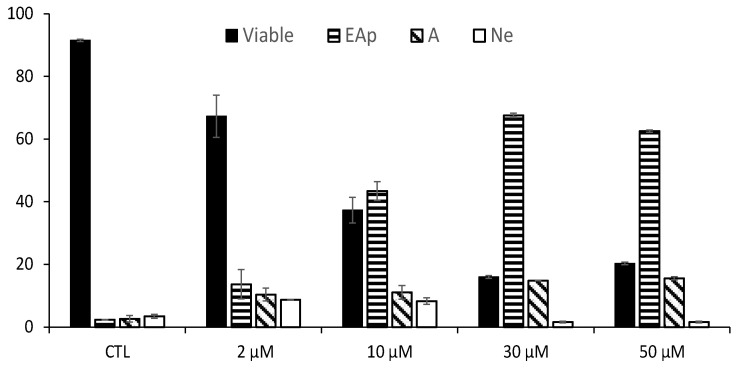
Effect of RM-581 on viable, early apoptotic (EAp), late apoptotic (LAp), and necrotic (Ne) LAPC-4 cells. The cells (3 × 10^5^) were exposed to RM-581 for 72 h at different concentrations (2, 10, 30, and 50 µM), and flow cytometric analysis using annexin-V and propidium iodide dyes allowed for the determination of each cell type. The experiment was performed in triplicate (mean ± SD).

The effect of RM-581 on LAPC-4 cell death was addressed by flow cytometry to determine the number of each cell type (Figure 4). A significant and increasing proportion of early and late apoptotic cells was observed according to the concentration (from 2 to 50 µM of RM-581). Thus, a major proportion of the cells entered the apoptotic stage at 30 and 50 µM, whereas viable cells dropped from 92% to 20%. At these two concentrations, the presence of necrotic cells is very low and non-significant.

### 3.2. RM-581 Blocks Tumor Growth in LAPC-4 Xenografts

**Figure 5:**
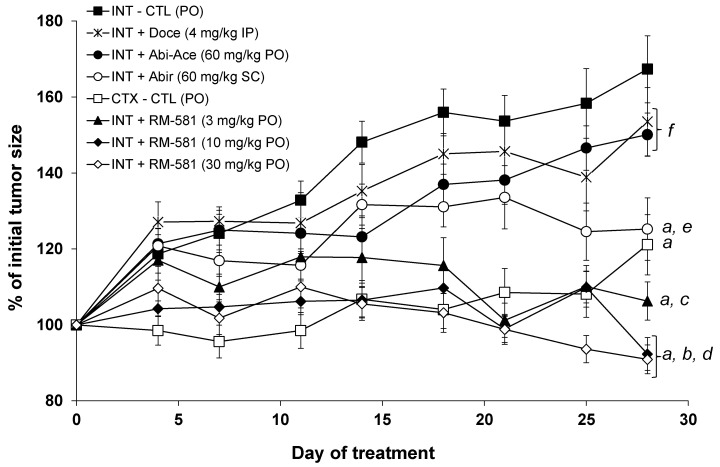
RM-581 blocks tumor growth in male immunosuppressed mice (LAPC-4 xenograft model). Effects of RM-581 (3, 10, and 30 mg/kg/PO/6 days per week), abiraterone acetate (60 mg/kg/PO/6 days per week), abiraterone (60 mg/kg/SC/6 days a week), and docetaxel (4 mg/kg/IP/twice a week). Tumor sizes are expressed as a percentage of the initial tumor size (day 1 of treatment: 100%). Data are the mean ± SEM (n = 16–20 tumors and 9–10 mice per group). *a*: *p* < 0.01 vs. INT-CTL; *b*: *p* < 0.01 vs. CTX-CTL; *c*: *p* < 0.05 vs. INT-Abir; *d*: NS vs. 10 and 30 mg/kg groups; *e*: NS vs. CTX-CTL; *f*: NS vs. INT-CTL.

Tumorigenic human LAPC-4 cells were inoculated in nude male mice. After 14 days, the mice with the generated tumors were randomized into different groups before starting each treatment (Figure 5). In this cancer model, the tumor size of the intact mice receiving only the vehicle (PG:DMSO/92:8) increased from 100% to 170% after 28 days, whereas the tumors of the mouse-castrated group did not increase until day 25, but a small non-significant increase was observed at day 28. As positive controls for treated groups, three known cytotoxic drugs, docetaxel, abiraterone, and abiraterone acetate, were tested. The first drug, docetaxel, was administered intraperitoneally (IP) twice a week at a dose of 4 mg/kg. During a previous xenograft with PC-3 cells [22], this dose of docetaxel made it possible to block tumor growth by 35% without significantly affecting the viability of the mice throughout the protocol. For LAPC-4 xenografts, however, no significant effect was observed for the dose of docetaxel, while the observed signs of toxicity (weight loss and swelling of the abdomen) prevented us from increasing the dose of docetaxel or its frequency of administration. For the other drugs, 60 mg/kg of abiraterone was administered subcutaneously (SC), while its prodrug, abiraterone acetate (60 mg/kg), was administered orally by gavage (PO). When used PO, abiraterone acetate showed a significant effect compared to the intact group after 14 days, but this effect was no longer significant at the end of the protocol (day 28). When administered at the same dose via the SC route, the CYP17A1 inhibitor abiraterone showed a significantly different effect compared to the intact control group on day 18. This effect continued to increase thereafter, reaching a 40% reduction by the end of the protocol (day 28).

Administered orally (PO) at 30 and 10 mg/kg, the aminosteroid RM-581 has been shown to be effective in rapidly blocking the growth of tumors at their starting levels (Figure 5). For the highest dose (30 mg/kg), a constant reduction in the size of the tumors is also observed from day 12, reaching an 88% reduction in the initial tumor size at the end of the protocol (day 28). For the median dose of RM-581 (10 mg/kg), the results were similar to those obtained at 30 mg/kg and identical at the end of the protocol. The lowest dose of RM-581 (3 mg/kg) also showed efficacy in blocking tumor growth, but tumors returned to their original size after 22 days. For the three groups treated with RM-581, a significant difference with the group of intact mice (untreated) was rapidly observed after 7, 7, and 14 days for the doses of 30, 10, and 3 mg/kg, respectively.

The concentration of RM-581 in plasma and tumors at the end of the protocol (3 h post-administration) was measured for the three treated groups of mice receiving the aminosteroid derivative orally (by gavage) (Table 2). Thus, the concentration of RM-581 increases from 15 to 171 ng/mL in plasma and from 50 to 940 ng/g in tumors, depending on the dose administered. Assuming an equivalence between ng/mL and ng/g, concentration index values of 3.3, 10, and 5.5 were calculated in the tumor depending on the dose administered (3, 10, and 30 mg/kg). When we estimated the concentration of RM-581 in the tumor and compared it to the IC_50_ of RM-581 in cells (~0.5 µM), the RM-581 concentration in the tumor was about three times higher for the 30 mg/kg dose, equivalent to the 10 mg/kg dose, and approximately six times lower for the 3 mg/kg dose. Despite the low concentration of RM-581 in the tumor at this last dose, RM-581 still stopped tumor growth, but this took longer than for the 10 and 30 mg/kg doses.

### 3.3. Assessment of RM-581 Toxicity

**Figure 6:**
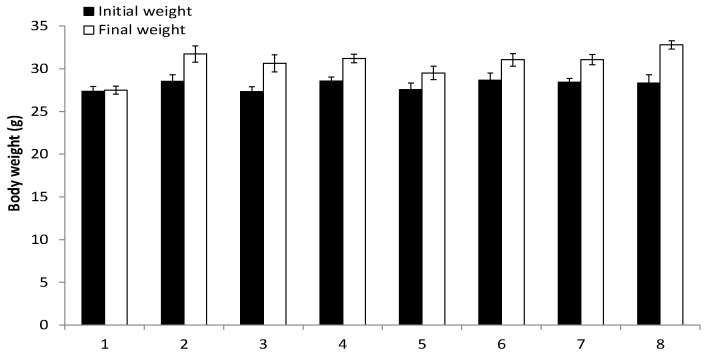
Body weight of mice before and after treatment (28 days). Mice were treated as in Figure 5. Gr-1: Control (CTL), Castrated (CTX) mice, PO; Gr-2: CTL, Intact mice, PO; Gr-3: Intact mice, RM-581 (30 mg/kg), PO; Gr-4: Intact mice, RM-581 (10 mg/kg), PO; Gr-5: Intact mice, RM-581 (3 mg/kg), PO; Gr-6: Intact mice, Abiraterone (60 mg/kg), SC; Gr-7: Intact mice, Abiraterone Acetate (60 mg/kg), PO; Gr-8: Intact mice, Docetaxel (4 mg/kg), IP.

As previously observed in xenograft protocols for other cancers (breast, pancreas, and prostate AR^−^) [18,21,22], no signs of apparent toxicity were observed for the duration of the LAPC-4 xenograft protocol (28 days). As another marker, there was no significant difference in the body weight of treated and untreated mice (Figure 6). As expected, a slight weight gain was observed in each of the groups between the beginning and the end of the protocol. Three tissues (liver, kidney, and prostate) of mice treated with RM-581 were also assessed by histology (hematoxylin and eosin staining), and their examination by a pathologist revealed that tissue morphology was not affected by the treatment. Thus, for the liver, there was no necrosis, steatosis, fibrosis, or cirrhosis; for the kidneys, there was an absence of tubular necrosis and glomerular fibrosis; and for the prostate, there were no histological lesions.

**Figure 7:**
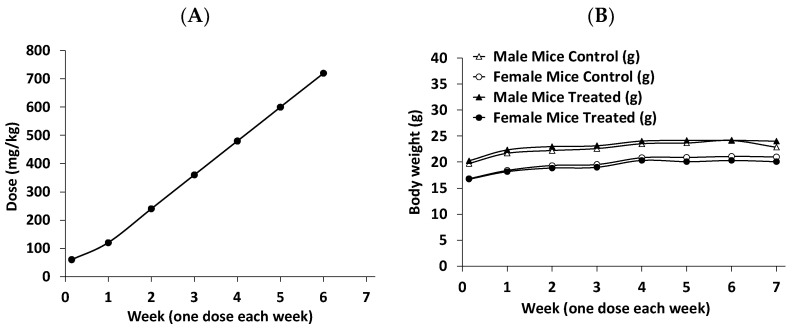
Determining the maximum tolerated dose of RM-581 in normal (not immunosuppressed) mice. Each week, an increased dose of RM-581 was given to each mouse (5 male mice/group and 5 female mice/group), and the signs of toxicity were monitored daily. The two control groups received only the vehicle (PG:DMSO/92:8). (**A**) Representation of the RM-581 doses (60, 120, 240, 360, 480, 600, and 720 mg/kg) administered orally by gavage (PO) per week. (**B**) Body weights of male and female mice receiving RM-581 or not (control) each week for 7 weeks.

RM-581 was also well tolerated by mice receiving three different doses (3, 10, and 30 mg/kg) PO, but to have an overview of its therapeutic index, the maximum tolerated dose was assessed in mice. Thus, normal mice (not immunosuppressed, contrary to the nude mice needed for xenograft experiments) received an increased oral dose of RM-581 each week (Figure 7A) and were observed daily for the various signs of toxicity approved by the ethics committee. According to animal behavior, no signs of toxicity were observed in the four different groups (males or females receiving 60, 120, 240, 360, 480, 600, and 720 mg/kg of RM-581 or only the vehicle). Additionally, no significant body weight difference was observed between the treated and control groups (Figure 7B). Considering the effective oral doses of RM-581 for tumor regression, 3–30 mg/kg in LAPC-4 xenograft (this study) and 60 mg/kg in PC-3 xenograft experiments [22], these data suggest a very good safety/tolerability profile for RM-581. Assuming effective doses of 10 and 30 mg/kg, the therapeutic index would be over 72 and 24 times. Thus, such a favorable safety profile of RM-581, even at high doses, should provide an advantageously flexible therapeutic window for future evaluation of RM-581 in humans.

### 3.4. Cholesterol and Fatty Acid Content Following RM-581 Treatment in LAPC-4 Xenografts

Lipids are recognized as important players in PC development and progression [40], which is why the impact of RM-581 on these key molecules was assessed. Thus, the concentrations of cholesterol and fatty acids were measured in different tissues (tumor, liver, and plasma) from the LAPC-4 xenograft experiment. Using a commercially available kit [28], total and free cholesterol were first measured in the tumor, liver, and plasma. As a result, a non-significantly different variation was found between the group treated with RM-581 (10 mg/kg) and the control group receiving only the vehicle (Table 3). The total cholesterol in plasma was also measured at the Medical Laboratory of the CHU de Québec/L’Enfant-Jésus Hospital. The concentrations obtained by the two methods were similar but slightly lower for the second method. There was no significant difference between the treated group (10 mg/kg) and the control group. The second assay method also made it possible to measure the concentrations of the different forms of cholesterol present in the plasma, namely triglycerides, cholesterol-HDL, cholesterol-LDL, cholesterol non-HDL, and the ratio of total cholesterol/cholesterol-HDL. However, no significant difference was observed between the treated mice and those in the control group (Figure S3, Supplementary Materials).

RM-581 has been shown to induce endoplasmic reticulum (ER) stress in different cancer cells and tumors leading to apoptosis [21,22,41]. Since ER stress has an impact on lipid homeostasis [42,43], the concentrations of total (T-FA), saturated (S-FA), and unsaturated (U-FA) fatty acids (FA) were measured in tumor, liver, and plasma (Table 4). The analysis includes a large panel of 56 FA determined by GC-FID in the 10 mg/kg group treated with RM-581 and the control group treated only with the vehicle. In tumors, the concentration of T-FA increased by 22.5%, but it increased more for the U-FAs (28.0%) than for the S-FAs (12.6%). The same trends were also observed in the liver. In plasma, on the other hand, the differences were not significant between the two groups analyzed. As expected, FA levels were much lower in plasma than in tissue. In Table 5, the characteristics and contents of the 17 FA most present and whose abundance was reported varied from 20.8% to 0.52%. In tumors treated with RM-581, the three most abundant FAs, which total 54.5% of T-FAs, increased by 16, 34, and 56%, respectively, for palmitic, oleic, and linoleic acids. Significant increases of 52, 12, 25, and 64% were also observed for palmitoleic, dihomo-γ-linolenic, myristic, and alpha-linolenic acids, but their abundances are lower (4.23, 2.35, 1.12, and 0.94%). Of the 17 FAs, only the content of adrenic acid-2 decreased significantly (−12%), but its abundance is low (1.1%).

### 3.5. RM-581 Treatment in LAPC-4 Cells (Genes and Cholesterol)

Previous work pointed out a disturbance of cholesterol homeostasis in PC-3 cells treated with RM-581 [22]. Consequently, the expression levels of several genes involved in different pathways (A-E) of biosynthesis and lipid action (cholesterol and fatty acids) were measured in LAPC-4 cells treated for 6, 18, 24, and 36 h with 5 μM of RM-581 (Table 6). For comparison, atorvastatin (10 μM), an inhibitor of HMGR, an enzyme involved in cholesterol synthesis, was tested as a control. Treatment of cells with atorvastatin induces different genes, including *ACLY* (involved in cholesterol synthesis and FA), *HMGCS1*, *HMGCR*, *MVK,* and *MVD* (involved in cholesterol synthesis), *FASN* and *SCD1* (involved in FA synthesis), as well as *LDLR* (involved in lipid transport). However, this treatment did not affect *ACAT1*, which is involved in cholesterol esterification. Treatment with RM-581 does not affect *ACLY* and *ACAT1* genes but affects genes in the B (except *MVK*), D, and E pathways. Total cholesterol in LAPC-4 cells treated for 18, 36, or 72 h with RM-581 (2 μM) or atorvastatin (10 μM) did not show a significant difference with untreated cells (Table S2, Supplementary Materials).

## 4. Discussion

The aminosteroid RM-581 emerged recently as a promising small molecule with anticancer action on a large spectrum of cell lines, including those refractory and/or resistant to the current chemotherapeutic arsenal [19,20,21,22]. Although the 5-year survival rate of PC is high, this cancer is, unfortunately, one of the most common, often evolving into forms that are resistant to known treatments. The arrival of a new drug could have a major impact on a high-fatality disease, such as metastatic PC, where the 5-year survival rate is only ~30%.

In addition to its antiproliferative activity in the low micromolar range against several cancer cell lines, including androgen-independent and androgen-dependent PC cell lines, RM-581 has been shown to be highly active (IC_50_~0.5 μM) on the AR^+^ metastatic LAPC-4 cell line used in the present study. Notably, its antiproliferative activity was found to be superior to that of enzalutamide (a new generation of antiandrogen) [44] and abiraterone (an inhibitor of androgen biosynthesis) [45], two drugs with which RM-581 showed a synergistic antiproliferative effect. Those results suggest that its action would not be directly linked to the hormonal pathway of androgens, as expected, since RM-581 was active on the two AR^−^ cell lines tested (PC-3 and DU-145). This is particularly interesting since the antiproliferative potency of abiraterone acetate or enzalutamide is relatively low (IC_50_ over 10 µM) in androgen-dependent and androgen-independent PC cell lines [46,47]. In the cell proliferation test, RM-581 was also more effective than docetaxel, a cytotoxic agent established in the last decade as the gold standard therapy for metastatic castration-resistant PC cases [48], producing an overall survival advantage of approximately three months [38]. Indeed, approximately half of the castration-resistant PC patients treated with docetaxel initially do not respond, and the second half ultimately develop resistance to this antineoplastic agent [49]. Thus, RM-581 would be an interesting addition to actual PC therapeutics to potentiate their action.

When tested in vivo (in the LAPC-4 xenograft tumor model), RM-581 administered orally by gavage (PO) was found to be more active than docetaxel administered IP, abiraterone administered SC, and abiraterone acetate administered PO. During this study, we also observed an accumulation of RM-581 in the tumors compared to plasma (3.3, 10, and 5.5 folds), which explains the better results obtained at the highest doses of 10 and 30 mg/kg. An interesting point observed several times for RM-581 is its innocuity during xenograft experiments from several types of cancers and duration of treatment (~1 month), which suggests a selectivity of action for cancer cells (tumors). This non-toxicity profile for nude mice was also confirmed in normal mice (immune system not suppressed) by carrying out a study where the dose of RM-581 administered orally increased each week, up to 720 mg/kg. Considering that the aminosteroid quickly produced an antitumor effect at a dose of 10 mg/kg, a high therapeutic index of over 70 was then obtained in mice.

At the end of the LAPC-4 xenograft protocol, the level of fatty acids (FA) had increased significantly in the tumor and liver of mice treated with RM-581 but not in the plasma. In these two tissues, the increase was greater in unsaturated FA (28 and 21%, respectively) than in saturated FA (7 and 11%, respectively). Some FA were also more affected than others, particularly saturated palmitic acid (+16%), monounsaturated oleic acid (+34%), and di-unsaturated linoleic acid (+56%), i.e., the 3 most abundant FA, with a total of 59% of the 56 FA measured. Interestingly, lipotoxicity was reported for oleic and linoleic acids in androgen-dependent PC cells [50,51]. Considering that an important increase in their level was observed inside the tumors (1.26 mg/g; ~4.5 mM and 1.59 mg/g; ~5.7 mM, respectively for oleic and linoleic acids), these lipids could thus contribute to the antitumoral effect of RM-581.

The level of cholesterol was not found to be significantly different in the tumor, liver, or plasma of mice treated or not with RM-581. Thus, although previous work pointed out an imbalance in cholesterol homeostasis caused by using RM-581 in the PC-3 cell line [22], this is not reflected in the level (increase or decrease) of cholesterol in the LAPC-4 xenograft experiment. The expression of several genes involved in the lipid-signaling pathway was also analyzed. Treatment of LAPC-4 cells with RM-581 increased the expression of genes involved in the synthesis of cholesterol (*HMGCS1*, *HMGCR,* and *MVD*), fatty acids (*SCD1*), and the LDL receptor (*LDLR*). This result is similar to that obtained when LAPC-4 cells were treated with atorvastatin, an inhibitor of HMGCR, an enzyme involved in cholesterol biosynthesis. Unlike atorvastatin, RM-581 did not increase *ACLY*, the gene that expresses a protein at the junction of both cholesterol and fatty acid synthesis.

Another difference was the expression of *FASN*, which was increased by the treatment of LAPC-4 cells with atorvastatin but decreased by treatment with RM-581. Therefore, the behavior of RM-581 is different from that of statins. Moreover, RM-581 led to a significant decrease in both FA and cholesterol in tumors from PC-3 xenografts [22], while it caused an increase in FA in tumors from LAPC-4 xenografts. This suggests that the impact of RM-581 on lipids appears to be different in androgen-independent (PC-3 cells) and androgen-dependent (LAPC-4 cells) PC xenografts. In their study, Furuya et al. [52] also showed the different effects of statins depending on the AR^−^ and AR^+^ status of the PC cells. In this study, the statin simvastatin decreased *LDLR* expression as well as the intracellular cholesterol ratio in PC-3 (AR^−^) cells. However, it increased *LDLR* expression without affecting the intracellular cholesterol ratio in LNCaP (AR^+^) cells. This last result is identical to that obtained by RM-581 on LAPC-4 cells. One explanation would be that PC (AR^+^) cells, such as LAPC-4 and LNCaP, would have an LDLR feedback mechanism [53] that would not be observed in AR^−^ cell lines, such as PC-3 and DU-145.

Two main hypotheses could explain the impact of RM-581 on the lipid metabolism seen in LAPC-4. The first one is that RM-581 induces a change in lipid metabolism as the first event, mainly on FA. This alteration subsequently provokes ER stress and UPR, which ultimately lead to apoptosis. In that case, the lipid metabolism change induced by RM-581 would be the cause of ER stress. In a second hypothesis, RM-581 rather initially induces ER stress and subsequent UPR, which leads to a change in lipid metabolism. In that situation, the lipid metabolism change would be a consequence of the ER stress induced by RM-581. Both hypotheses are plausible since the alteration of lipid levels is known to induce ER stress in cancer cells, and ER stress is also known to disturb lipogenesis [43,54]. Considering that it has been previously demonstrated in various cancer cell lines using RM-581-Fluo as a fluorescent probe that RM-581 specifically accumulates in the endoplasmic reticulum (ER) [22,41], the substantial influx of an exogenous compound into the ER membrane could potentially disrupt crucial molecular components, leading to ER-related apoptosis. It is also possible that a disturbance of FA levels contributes to exacerbating ER stress and creating a kind of vicious circle, overriding UPR resolution capacity.

As stated above, after treatment with RM-581, the levels of lipids decreased in PC-3 tumors, contrary to LAPC-4, where an increase in lipid levels was measured. These opposite results in those two PC subtypes suggest that ER stress takes place independently of the change in lipid levels, triggering UPR and subsequent apoptosis. Despite this, it is not impossible that lipid variations could ultimately lead to apoptosis, which would support the hypothesis that RM-581 first provokes ER stress and then has an impact on lipid levels, considering that a rather low amplitude of lipid variation is measured in both PC-3 and LAPC-4 tumors. In fact, it would be rather surprising that such a relatively low variation (~20%) could be solely responsible for the cytotoxicity observed for RM-581 in the submicromolar range (IC_50_ = 0.5 µM) considering that statins, which have a stronger impact on lowering cholesterol levels, are less cytotoxic to PC cells (IC_50_ > 10 µM) [55]. Nevertheless, while the observed lipid variation in LAPC-4 cells can contribute to the overall ER stress phenomenon to some extent, it appears more plausible to support the hypothesis that the primary event triggering apoptosis in PC cells is primarily caused by a detrimental disturbance of ER homeostasis induced by RM-581.

## 5. Conclusions

In studies with metastatic LAPC-4 androgen-dependent PC cells, the aminosteroid RM-581 showed promising anticancer effects both in vitro and in vivo. The most important result is its ability to completely block tumor growth (LAPC-4 xenografts) in immunosuppressed mice when administered orally at a dose as low as 3 mg/kg. Another important result is the innocuity of RM-581 during the xenograft experiment carried out for 28 days in immunosuppressed mice, as well as during a dose-escalation study in non-immunosuppressed male and female mice whose last dose of RM-581 was 720 mg/kg, administered orally. In fact, we did not observe an inflammatory response after drug administration. Given the biological results obtained with RM-581, an aminosteroid that induces ER stress leading to apoptosis of cancer cells, and the fact that it is easily prepared using short chemical synthesis, this steroid derivative is a promising candidate for clinical trials on resistant PC patients when used alone or in combination with actual gold standard drugs.

## Figures and Tables

**Table 1:** Viability screening experiments with RM-581 on various prostate cancer (PC) cell lines.

Entry	PC Cell Lines	AR Status ^1^	IC_50_ (μM) ^2^
1	DU-145	N	4.4 [22]
2	PC-3	N	1.2 [22]
3	LNCaP	P	1.2 [22]
4	VCaP	P	5.78
5	22Rv1	P	1.38
6	LAPC-4	P	0.56

^1^ Androgen receptor (AR) status as negative (N) or positive (P). ^2^ Cell proliferation was assessed after three days of treatment using MTS (entries 1–3, 5, and 6) or seven days (entry 4). For entries 4–6, the results of one representative experiment out of 2–4 independent experiments performed with eight replicates are shown. See Figure S1, Supplementary Materials.

**Table 2:** Tumor and plasma concentrations of RM-581 from LAPC-4 xenograft experiment.

RM-581 Dose (mg/kg) ^1^	RM-581 in Tumor (ng/g—*µM*) ^2^	RM-581 in Plasma (ng/mL—*µM*) ^2^	Concentration Index (Tumor/Plasma) ^3^
3	50—*0.077*	15—*0.023*	3.3
10	300—*0.464*	30—*0.046*	10
30	940—*1.465*	171—*0.265*	5.5

^1^ RM-581 was administered orally by gavage (PO). ^2^ Tumor and plasma were calculated 3 h after the last treatment, stored at −80 °C until the concentration of RM-581 was measured by LC-MS/MS analysis. ^3^ For the tumor concentration of RM-581, we estimated ng/g = ng/mL.

**Table 3:** Tumor, liver, and plasma cholesterol concentrations (µg/g or µg/mL) were obtained from LAPC-4 xenografts in mice (CTL and 10 mg/kg groups).

Tissue	Method ^1^	T-CHOL CTL	T-CHOL RM-581 (10 mg/kg)	F-CHOL CTL	F-CHOL RM-581 (10 mg/kg)
Tumor (µg/g)	A	4453 ± 630	4543 ± 2476 (NS)	3610 ± 354	3270 ± 236 (NS)
Liver (µg/g)	A	2930 ± 67	2440 ± 475 (NS)	2930 ± 408	2410 ± 10 (NS)
Plasma (µg/mL)	A	1700 ± 200	1820 ± 500 (NS)	370 ± 40	403 ± 120 (NS)
Plasma (µg/mL)	B	1320 ± 167	1182 ± 338 (NS)	--	--

^1^ Methods A (commercially available kit [28]) and B (Medical Laboratory of CHU de Québec/L’Enfant-Jésus Hospital). T-CHOL: Total cholesterol; F-CHOL: Free cholesterol. NS: not significantly different from control (CTL).

**Table 4:** Tumor, liver, and plasma fatty acid concentrations (mg/g or μg/mL) were obtained from LAPC-4 xenografts in mice (CTL and 10 mg/kg groups).

Tissue	T-FA CTL	T-FA RM-581 (10 mg/kg)	%	S-FA CTL	S-FA RM-581 (10 mg/kg)	%	U-FA CTL	U-FA RM-581 (10 mg/kg)	%
Tumor (mg/g)	20.0	24.5	+22.5	7.24	8.15	+12.6	12.5	16.0	+28.0
Liver (mg/g)	28.8	34.5	+19.8	10.6	12.5	+17.9	18.1	21.9	+21.0
Plasma (µg/mL)	3.65	3.71	+1.6	1.07	1.10	+2.8	2.55	2.59	+1.6

RM-581 (10 mg/kg) was administered orally (gavage) in mice for 28 days of treatment. Control (CTL) mice received only the vehicle (PG:DMSO/92:8). The concentrations of 56 fatty acids (FA) were measured by GC-FID in duplicate. T-FA: Total of 56 fatty acids; S-FA: Saturated fatty acids; U-FA: Unsaturated fatty acids.

**Table 5:** Fatty acid content in tumors treated with RM-581 (10 mg/kg) or not (CTL) from LAPC-4 xenografts in mice.

Figure ^1^		FA (Double Bond)	% of Total FA	FA CTL (mg/g)	FA RM-581 (mg/g)	%
Palmitic acid	-	16:0	20.8	4.395	5.095	+16
Oleic acid (9c)	w9	18:1	20.1	3.665	4.920	+34
Linoleic acid (9c12c)	w6	18:2	18.1	2.840	4.435	+56
Stearic acid	-	18:0	9.61	2.275	2.355	+3.5
Arachidonic (5c8c11c14c)	w6	20:4	8.77	2.195	2.150	−2.1
Palmitoleic acid (9c)	w7	16:1	4.23	0.680	1.035	+52
Vaccenic acid (11c)	w7	18:1	2.82	0.655	0.690	+5.3
Dihomo-γ-linolenic acid (8c11c14c)	w6	20:3	2.35	0.520	0.580	+12
Cervonic acid (4c7c10c13c16c19c)	w3	22:6	2.18	0.530	0.530	0
Adrenic acid-1 (7c10c13c16c)	w6	22:4	1.54	0.350	0.380	+8.6
Myristic acid	-	14:0	1.12	0.220	0.275	+25
Adrenic acid-2 (4c7c10c13c16c)	w6	22:5	1.07	0.295	0.260	−12
Alpha-linolenic acid (9c12c15c)	w3	18:3	0.94	0.140	0.230	+64
Dimethoxyhexadecanoic acid	-	16:0	0.68	0.170	0.170	0
Lignoceric acid	-	24:0	0.65	0.130	0.160	+23
Docosapentaenoic acid (7c10c13c16c19c)	w3	22:5	0.57	0.120	0.140	+17
Nervonic acid (15c)	w9	24:1	0.52	0.110	0.130	+18

^1^ RM-581 (10 mg/kg) was administered orally (gavage) in mice for 28 days of treatment. Control (CTL) mice received only the vehicle (PG:DMSO/92:8). Only the 17 most abundant (>0.5%) fatty acids (FA) were listed from the 56 whose concentration was measured by GC-FID in duplicate (Table S1, Supplementary Materials).

**Table 6:** RM-581 triggers in LAPC-4 cells the mRNA expression of various genes involved in cholesterol and fatty acid pathways ^1^.

Gene	Path-Way	RM-581 6 h	RM-581 18 h	RM-581 24 h	RM-581 36 h	Athor 6 h	Athor 18 h	Athor 24 h	Athor 36 h
*ACLY*	A	0.8	0.4 **	0.9	0.9	1.0	1.2	1.9 *	2.2 *
*HMGCS1*	B	2.7 **	3.3 **	2.4 **	2.0 **	1.3	3.5 *	2.7 *	2.0 *
*HMGCR*	B	1.9 **	1.2	2.6 **	2.5 **	1.4 *	1.8 *	2.6 *	3.5 *
*MVK*	B	1.2	1.6	1.0	1.0	1.5	2.0 *	2.5 *	2.5 *
*MVD*	B	2.7 **	1.8 **	2.1 **	1.6 **	1.6 *	2.6 *	3.5 *	4.0 *
*ACAT1*	C	0.7 *	0.9	1.1	1.2	1.0	1.0	1.0	1.1
*FASN*	D	1.0	0.5 **	0.6 *	0.6 *	1.2	1.3	1.7 *	1.8 *
*SCD1*	D	0.7	0.8	2.5 **	3.5 **	1.4	1.6 *	2.6 *	4.5 *
*LDLR*	E	1.4	1.8 **	2.6 **	2.5 **	1.0	1.8 *	1.8 *	2.5 *

^1^ LAPC-4 cells were treated with RM-581 (5 µM) or atorvastatin (10 µM) and mRNA expression was determined at 6, 18, 24, and 36 h post-treatment. Pathways: (A) Cholesterol and fatty acid syntheses; (B) cholesterol synthesis; (C) cholesterol and fatty acid esterification; (D) fatty acid synthesis; and (E) low-density lipoprotein receptor. Each data point represents the mean of an experiment performed in triplicate (mean ± SD). * *p* < 0.05, ** *p* < 0.01 vs. the control (CTL). Basal expression of genes in the CTL is fixed at 1.0.

## Data Availability

Data are contained within the article and Supplementary Materials.

## Supplementary Materials

The following supporting information can be downloaded at: https://www.mdpi.com/article/10.3390/cancers15113033/s1, Figure S1: Efficacy of RM-581 in reducing the LAPC-4, 22Rv1 and VCaP cell proliferation after 3 days of treatment (LAPC-4 and 22Rv1) or 7 days of treatment (VCaP); Figure S2: Efficacy of docetaxel and RM-581 in reducing LAPC-4 cell proliferation after 6 days of treatment; Figure S3: Concentrations in μg/mL or mmol/L of total cholesterol, triglycerides, cholesterol-HDL, cholesterol-LDL, cholesterol non-HDL and ratio of total cholesterol/cholesterol-HDL obtained from LAPC-4 xenograft experiments with RM-581 (3, 10 and 30 mg/kg/PO) and abiraterone acetate (60 mg/kg/PO); Table S1: Content of 56 fatty acids detected in tumors treated with RM-581 (10 mg/kg) or not (CTL) from LAPC-4 xenograft experiment in mice; Table S2: Total cholesterol concentration in LAPC-4 cells treated 18, 36 and 72 h with the aminosteroid RM-581 (2 μM) or atorvastatin (10 μM); and Table S3: Primer sequences for different genes.
