# Supplementary material for: An Aminosteroid Derivative Shows Higher In Vitro and In Vivo Potencies than Gold Standard Drugs in Androgen-Dependent Prostate Cancer Models
Source: Cancers (Basel). 2023 Jun 2;15(11):3033. doi: 10.3390/cancers15113033 (PMC10252594; doi:10.3390/cancers15113033)
Supplement: Supplementary file 1 [file cancers-15-03033-s001.zip › cancers-2350311-SI.pdf]

Supplementary Materials

An Aminosteroid Derivative Shows Higher In Vitro and In Vivo Potencies than Gold Standard Drugs in Androgen-Dependent Prostate Cancer Models

Donald Poirier,[1,2,*] Jenny Roy,[1] René Maltais,[1] Cindy Weidmann,[1] Étienne Audet-Walsh[1,2]

[1] Endocrinology and Nephrology Unit, CHU de Québec Research Center-Université Laval, Pavillon CHUL, Québec, QC, G1V 4G2, Canada

[2] Department of Molecular Medicine, Faculty of Medicine, Université Laval, Québec, QC, G1V 0A6, Canada

Content: Figure S1, Figure S2, Figure S3, Table S1, Table S2 and Table S3

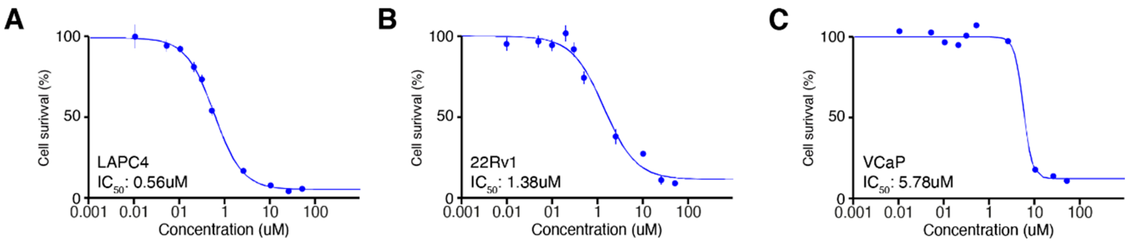

Figure S1. Efficacy of RM-581 in reducing the LAPC-4 (**A**), 22Rv1 (**B**) and VCaP (**C**) cell proliferation after 3 days of treatment (LAPC-4 and 22Rv1) or 7 days of treatment (VCaP). Results from one representative experiment out of 2-4 independent experiments performed with 8 replicates per conditions are shown. Concentration is shown in $\log_{10}$ scale.

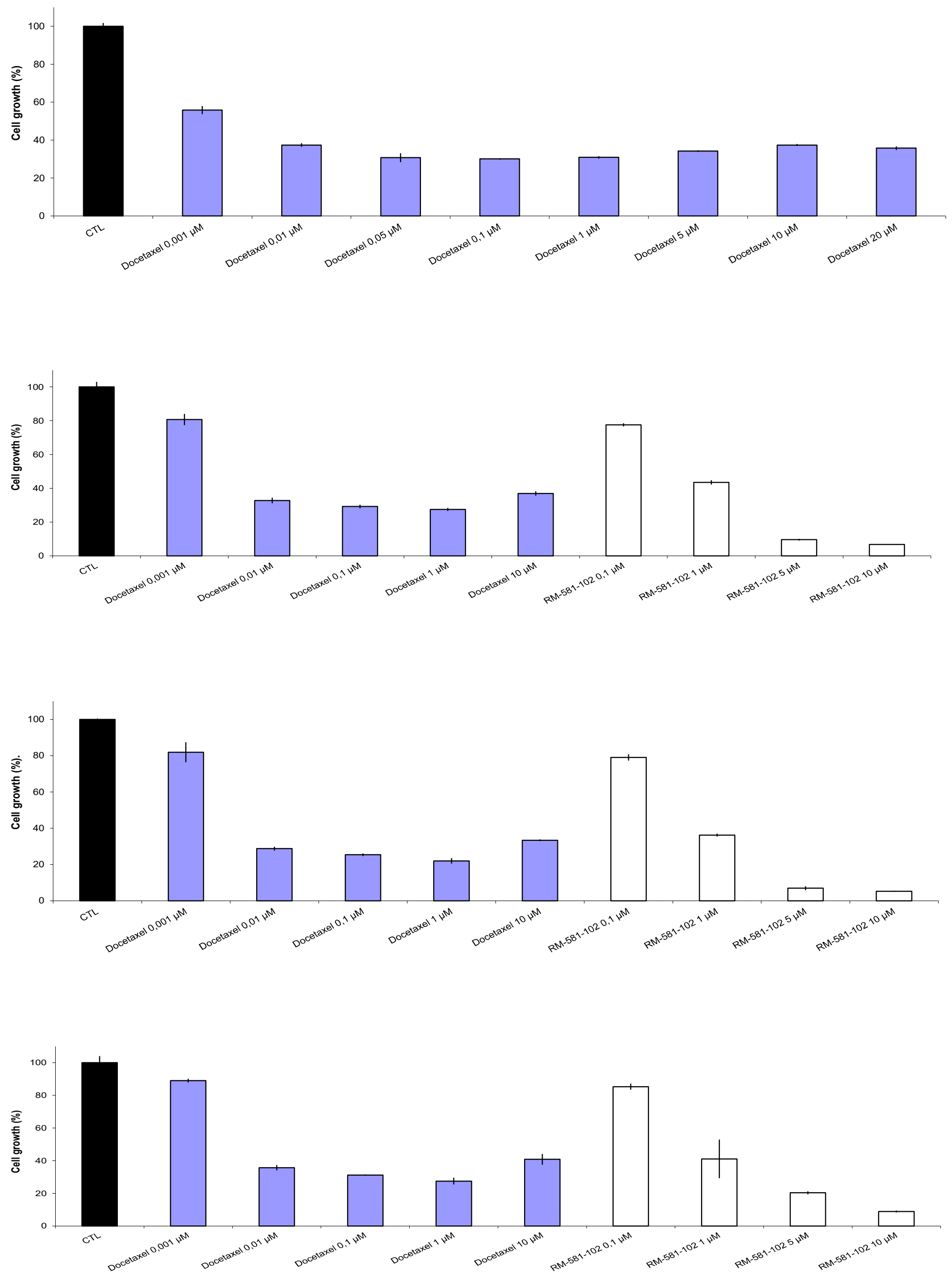

Figure S2. Efficacy of docetaxel and RM-581 in reducing LAPC-4 cell proliferation after 6 days of treatment. **A**) Standard cell culture protocol with change of medium, **B**) with an initial washing of the cells, **C**) with an initial washing of the cells and without change of medium, and **D**) without an initial washing of the cells and with change of medium. For docetaxel, results are the mean of 4 experiments performed in triplicate. For docetaxel, IC_{50} value was not calculated. For RM-581, results are the mean of 3 experiments performed in triplicate with IC_{50} = 0.51, 0.47 and 0.73 µM. Mean IC_{50} = 0.57 ± 0.14 µM.

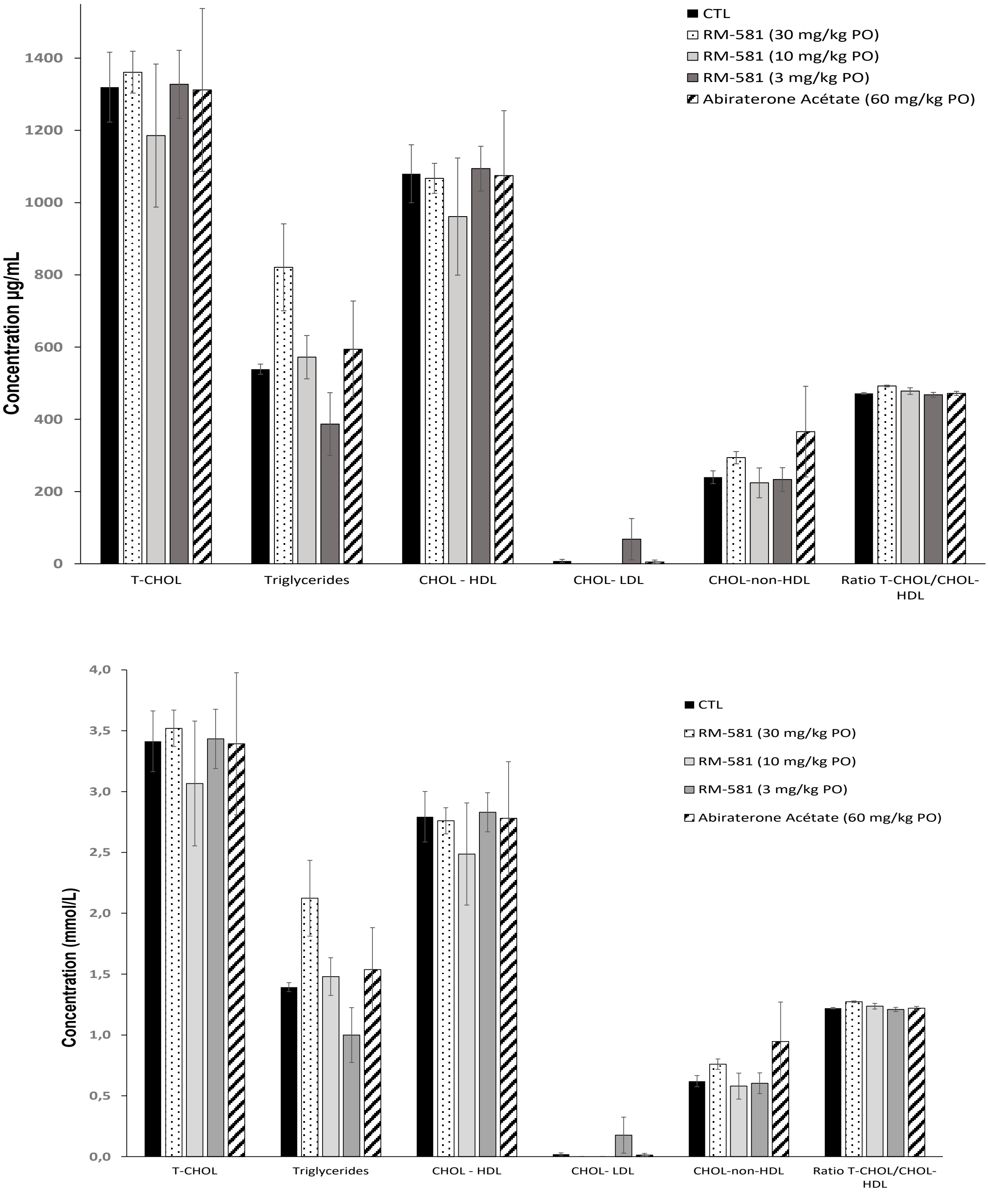

Figure S3. Concentrations in µg/mL (up) or mmol/L (down) of total cholesterol, triglycerides, cholesterol-HDL, cholesterol-LDL, cholesterol non-HDL and ratio of total cholesterol/cholesterol-HDL obtained from LAPC-4 xenograft experiments with RM-581 (3, 10 and 30 mg/kg/PO) and abiraterone acetate (60 mg/kg/PO). Method B (Medical Laboratory of CHU de Québec/L'Enfant Jésus Hospital).

Table S1. Content of 56 fatty acids detected in tumors treated with RM-581 (10 mg/kg) or not (CTL) from LAPC-4 xenograft experiment in mice.

**Fatty Acid (FA) Content**[1]	**Series**	**FA** (double bond)	**FA RM-581** % of total	**FA CTL** (mg/g)	**FA RM-581** (mg/g)	**%**[2]
More abundant FA (> 0.5%)						
Palmitic acid	-	16:0	20.8	4.395	5.095	+ ***16***
Oleic acid (9c)	w9	18:1	20.1	3.665	4.920	**+ 34**
Linoleic acid (9c12c)	w6	18:2	18.1	2.840	4.435	**+ 56**
Stearic acid	-	18:0	9.61	2.275	2.355	+ *3.5*
Arachidonic (5c8c11c14c)	w6	20:4	8.77	2.195	2.150	- 2.1
Palmitoleic acid (9c)	w7	16:1	4.23	0.680	1.035	**+ 52**
Vaccenic acid (11c)	w7	18:1	2.82	0.655	0.690	+ 5.3
Dihomo-γ-linolenic acid (8c11c14c)	w6	20:3	2.35	0.520	0.580	+ 12
Cervonic acid (4c7c10c13c16c19c)	w3	22:6	2.18	0.530	0.530	0
Adrenic acid-1 (7c10c13c16c)	w6	22:4	1.54	0.350	0.380	+ 8.6
Myristic acid	-	14:0	1.12	0.220	0.275	**+ 25**
Adrenic acid-2 (4c7c10c13c16c)	w6	22:5	1.07	0.295	0.260	- 12
Alpha-linolenic acid (9c12c15c)	w3	18:3	0.94	0.140	0.230	**+ 64**
Dimethoxyhexadecanoic acid	-	16:0	0.68	0.170	0.170	0
Lignoceric acid	-	24:0	0.65	0.130	0.160	+ 23
Docosapentaenoic acid(7c10c13c16c19c)	w3	22:5	0.57	0.120	0.140	+ 17
Nervonic acid (15c)	w9	24:1	0.52	0.110	0.130	+ 18
Less abundant FA (< 0.5%)						
Caprylic acid	--	8:0	0	0	0	--
Caproic acid	--	10:0	0.02	0	0	--
Lauric acid	--	12:0	0.10	0.02	0.02	--
-- (9t)	w5	14:1	0.03	0.01	0.01	--
Myristoleic acid (9c)	w5	14:1	0.06	0.01	0.02	--
Pentadecanoic acid	--	15:0	0.17	0.03	0.04	--
-- (5t)	w10	15:1	0	0	0	--
-- (5c)	w10	15:1	0	0	0	--
-- (1c)	w14	15:1	0	0	0	--
Palmitelaidic acid (9t)	w1	16:1	0	0	0	--
Isobranched	--	17:0	0.08	0.02	0.02	--
Dimethoxyoctadecanoic acid	--	18:0	0.38	0.075	0.09	--
-- (7t)	w10	17:1	0.09	0.02	0.02	--
Dimethoxyoctadecenoic acid (9c)	w9	18:1	0.29	0.055	0.07	--
-- (7c)	w10	17:1	0.20	0	0.05	--
Petroselaidic acid (6t)	w12	18:1	0	0	0	--
Elaidic acid (9t)	w9	18:1	0	0	0	--

Trans-vaccenic acid (11t)	w7	18:1	0.13	0.03	0.03	--
Petroselinic acid (7c-n11/6c-n12)	w11/12	18:1	0	0	0	--
-- (12c)	w6	18:1	0	0	0	--
-- (13c)	w5	18:1	0.07	0.02	0.02	--
Linolelaidic acid (9t12t)	w6	18:2	0.14	0.03	0.03	--
-- (9c12t)	w6	18:2	0.09	0.02	0.02	--
-- (9t12c)	w6	18:2	0	0	0	--
-- (9t12t15t)	w3	18:3	0.03	0.01	0.01	--
Arachidic acid	--	20:0	0.20	0.04	0.05	--
Gamma-linolenic acid (6c9c12c)	w6	18:3	0.19	0.04	0.05	--
-- (8c)	w12	20:1	0	0	0	--
Gondoic acid (11c)	w9	20:1	0.32	0.05	0.08	--
Stearidonic acid (6c9c12c15c)	w3	18:4	0	0	0	--
-- (11c14c)	w6	20:2	0.34	0.06	0.08	--
Behenic acid	--	22:0	0.35	0.07	0.09	--
-- (9t)	w13	22:1	0	0	0	--
-- (11c14c17c)	w3	20:3	0	0	0	--
Erucic acid	w9	22:1	0.10	0.02	0.025	--
-- (8c11c14c17c)	w3	20:4	0.16	0.015	0.04	--
-- (13c16c)	w6	22:2	0.04	0.01	0.01	--
Timnodonic acid (5c8c11c14c17c)	w3	20:5	0.10	0.02	0.025	--
-- (13c16c19c)	w3	22:3	0	0	0	--

[1] RM-581 (10 mg/kg) was administrated orally (gavage) in mice (28 days of treatment). Control (CTL) mice received only the vehicle (PG:DMSO/92:8). [2] Calculated only for the 17 most abundant fatty acids (> 0.5%) whose concentration was measured by GC-FDI.

Table S2. Total cholesterol concentration in LAPC-4 cells treated 18, 36 and 72 h with the aminosteroid RM-581 (2 µM) or atorvastatin (10 µM).

Time (h)	Control	RM-581 (2 µM)	Atorvastatin (10 µM)
18	65 ± 0.7	68 ± 3	59 ± 3.5
36	62 ± 1	69 ± 6	61 ± 0.4
72	66 ± 2	69 ± 4	70 ± 1
72	108 ± 6.5	--	50 ± 2

Table S3. Primer sequences for different genes

Gene	Name	Primer sequences
ACLY	ACLY_F	CGGACTTCGGCAGAGGTAGA
	ACLY_R	GTTGACCCCAACGAGACCAA
HMGCS1	HMGCS1_F	GTCACGCTTGTGCCCGAA
	HMGCS1_R	GCCGCCCAATGCAATCATAG
HMGCR	HMGCR_F	TTCGGTGGCCTCTAGTGAGA
	HMGCR_R	AAAGCTTCATTCAAGCCTGTCA
MVK	MVK_F	GAGCCATGTTGTCAGAAGTCC
	MVK_R	GTGACATCACCTTGCTCCAGAA
MVD	MVD_F	TCATCAAGTACTGGGGCAAGC
	MVD_R	GGTTTTTAACTGGTCCTGGTGC
ACAT1	ACAT1_F	GGAGGCTGGTGCAGGAAATA
	ACAT1_R	TCCTGATGTCCACACATAAGACT
FASN	FASN_F	ATGGAGGAGGTGGTGATTGC
	FASN_R	CTGGGCCCTCTGAAGTCGAA
SCD1	SCD1_F	AACAGCAGGAGCTCATCGTC
	SCD1_R	AAGTTGATGTGCCAGCGGTA
LDLR	LDLR_F	GAGGGCTCTGTCCATTGTCC
	LDLR_R	ACCATCTGTCTCGAGGGGTA
